# Virulence genes and previously unexplored gene clusters in four commensal *Neisseria* spp. isolated from the human throat expand the neisserial gene repertoire

**DOI:** 10.1099/mgen.0.000423

**Published:** 2020-08-26

**Authors:** Alan Calder, Chukwuma Jude Menkiti, Aylin Çağdaş, Jefferson Lisboa Santos, Ricarda Streich, Alice Wong, Amir H. Avini, Ebrima Bojang, Karththeepan Yogamanoharan, Nivetha Sivanesan, Besma Ali, Mariam Ashrafi, Abdirizak Issa, Tajinder Kaur, Aisha Latif, Hani A. Sheik Mohamed, Atifa Maqsood, Laxmi Tamang, Emily Swager, Alex J. Stringer, Lori A.S. Snyder

**Affiliations:** ^1^​ School of Life Sciences, Pharmacy, and Chemistry, Kingston University, Kingston upon Thames, KT1 2EE, UK

**Keywords:** *Neisseria subflava*, *Neisseria cinerea*, bacterial capsule, T6SS, natural competence for transformation

## Abstract

Commensal non-pathogenic *Neisseria* spp. live within the human host alongside the pathogenic *Neisseria meningitidis* and *Neisseria gonorrhoeae* and due to natural competence, horizontal gene transfer within the genus is possible and has been observed. Four distinct *Neisseria* spp. isolates taken from the throats of two human volunteers have been assessed here using a combination of microbiological and bioinformatics techniques. Three of the isolates have been identified as *Neisseria subflava* biovar *perflava* and one as *Neisseria cinerea *. Specific gene clusters have been identified within these commensal isolate genome sequences that are believed to encode a Type VI Secretion System, a newly identified CRISPR system, a Type IV Secretion System unlike that in other *Neisseria* spp., a hemin transporter, and a haem acquisition and utilization system. This investigation is the first to investigate these systems in either the non-pathogenic or pathogenic *Neisseria* spp. In addition, the *N. subflava* biovar *perflava* possess previously unreported capsule loci and sequences have been identified in all four isolates that are similar to genes seen within the pathogens that are associated with virulence. These data from the four commensal isolates provide further evidence for a *Neisseria* spp. gene pool and highlight the presence of systems within the commensals with functions still to be explored.

## Data Summary

Sequence data for commensal *Neisseria* spp. investigated are available in GenBank under the following accession numbers: *Neisseria subflava* strains M18660 (NZ_CP031251; https://www.ncbi.nlm.nih.gov/nuccore/NZ_CP031251.1); ATCC 49275 (NZ_CP039887; https://www.ncbi.nlm.nih.gov/nuccore/NZ_CP039887.1); NJ9703 (NZ_ACEO00000000; https://www.ncbi.nlm.nih.gov/nuccore/NZ_ACEO00000000.2); C2012011976 (NZ_POXP00000000; https://www.ncbi.nlm.nih.gov/nuccore/NZ_POXP00000000.1); C2011004960 (NZ_POXK00000000; https://www.ncbi.nlm.nih.gov/nuccore/NZ_POXK00000000.1); C2009010520 (NZ_POXD00000000; https://www.ncbi.nlm.nih.gov/nuccore/NZ_POXD00000000.1); C2011020198 (NZ_POXM00000000; https://www.ncbi.nlm.nih.gov/nuccore/NZ_POXM00000000.1); C2005001510 (NZ_POWU00000000; https://www.ncbi.nlm.nih.gov/nuccore/NZ_POWU00000000.1); C2014021188 (NZ_POYB00000000; https://www.ncbi.nlm.nih.gov/nuccore/NZ_POYB00000000.1); C2011020199 (NZ_POXN00000000; https://www.ncbi.nlm.nih.gov/nuccore/NZ_POXN00000000.1); C2011033015 (NZ_POXO00000000; https://www.ncbi.nlm.nih.gov/nuccore/NZ_POXO00000000.1); C2008002238 (NZ_POXC00000000; https://www.ncbi.nlm.nih.gov/nuccore/NZ_POXC00000000.1); C2011009653 (NZ_POXL00000000; https://www.ncbi.nlm.nih.gov/nuccore/NZ_POXL00000000.1); C2007002879 (NZ_POWV00000000; https://www.ncbi.nlm.nih.gov/nuccore/NZ_POWV00000000.1); C2008001664 (NZ_POXB00000000; https://www.ncbi.nlm.nih.gov/nuccore/NZ_POXB00000000.1); *Neisseria mucosa* strain FDAARGOS_260 (NZ_CP020452; https://www.ncbi.nlm.nih.gov/nuccore/NZ_CP020452.2); *Neisseria flavescens ATCC 13120* (NZ_CP039886; https://www.ncbi.nlm.nih.gov/nuccore/NZ_CP039886.1); *Neisseria cinerea* strain NCTC10294 (NZ_LS483369; https://www.ncbi.nlm.nih.gov/nuccore/NZ_LS483369.1). Sequence data for *Neisseria meningitidis* strains investigated are available in GenBank under the following accession numbers: MC58 (AE002098.2; http://www.ncbi.nlm.nih.gov/nuccore/AE002098.2); FAM18 (NC_008767.1; http://www.ncbi.nlm.nih.gov/nuccore/NC_008767.1); Z2491 (AL157959.1; http://www.ncbi.nlm.nih.gov/nuccore/AL157959.1). Sequence data for *Neisseria gonorrhoeae* strains investigated are available in GenBank under the following accession numbers: FA1090 (AEOO4969.1; http://www.ncbi.nlm.nih.gov/nuccore/AE004969.1); NCCP11945 (CP001050.1; http://www.ncbi.nlm.nih.gov/nuccore/CP001050.1). Sequence data for *Neisseria lactamica* strains investigated are available in GenBank under the following accession numbers: 020–06 (NC_014752.1; http://www.ncbi.nlm.nih.gov/nuccore/NC_014752.1). The authors confirm all supporting data, code and protocols have been provided within the article or through supplementary data files.

**Impact Statement**

To date, most research into *Neisseria* spp. has focused on the pathogens *Neisseria meningitidis* and *Neisseria gonorrhoeae *, but many commensal non-pathogens of this genus contain gene repertoires that are worthy of investigation, including those associated with virulence in the pathogens. Horizontal gene transfer (HGT) has been demonstrated between *Neisseria* spp. The sequences revealed in this investigation are therefore potential sources of genetic material for the pathogens via HGT. Acquisition of the capsule genes described here by *N. meningitidis* could result in capsule switching and enable circumvention of serogroup-specific vaccines. In combination with alleles for vaccine targets fHbp and NadA, potential exists for vaccine escape via HGT from these commensal genomes and other sequences in the circulating gene pool. The data presented here provide further evidence for a wide and varied *Neisseria* spp. gene pool and emphasize the presence of numerous ‘virulence genes’ within the commensal species, requiring the functions of these to be re-evaluated. This research additionally highlights the presence of previously unexplored systems within the commensal *Neisseria* spp. with functions still to be explored, which may lead to the development of novel therapeutic interventions with regard to pathogen-related infections.

## Introduction

The human oral, nasal, and pharyngeal cavities are inhabited by hundreds of different bacterial species, with the throat possessing a greater number than any other body site [1]. The most common isolates from this site in humans belong to the phyla Actinobacteria, Bacteroidetes, Firmicutes, Fusobacteria, Spirochaetes*,* and Proteobacteria, with Proteobacteria contributing to around 17 % of the overall microbiome [2]. Of the β-Proteobacteria, the family *Neisseriaceae* comprise Gram-negative coccoid bacteria with a preference for colonizing mucosal surfaces within the nasopharyngeal and oral cavities of humans [3]. These micro-organisms are considered to be a major constituent of the core microbiome of the oral cavity [2] and contribute significantly to the normal flora at these sites [3].

Overall the genus *Neisseria* is composed of a number of species, including two pathogens, *Neisseria meningitidis* and *Neisseria gonorrhoeae *, and non-pathogenic species such as *Neisseria lactamica *, *Neisseria cinerea *, *Neisseria elongata *, *Neisseria flavescens *, *Neisseria mucosa *, *Neisseria polysaccharea *, *Neisseria sicca *, *Neisseria weaveri *, *Neisseria animaloris *, *Neisseria bacilliformis *, *Neisseria zoodegmatis *, *Neisseria oralis *, *Neisseria canis *, *Neisseria shayeganii,* and *Neisseria subflava* biovars *flava*, *perflava,* and *subflava* [4]. Commensal *Neisseria* are normally harmless, although some have been identified as opportunistic pathogens and have caused rare cases of sepsis and meningitis [3, 5]. Virulence in *N. meningitidis* is mediated by a number of factors, including the expression of a range of adhesins, lipooligosaccharide endotoxins (LOS), a polysaccharide capsule for evasion of the host immune response, and iron acquisition systems [6]. While capsule genes are often associated with *N. meningitidis *, they have also been identified within the non-pathogenic *Neisseria* spp. [7].

Despite iron being essential [8], iron acquisition within a host is considered to be a major virulence determinant and vital to pathogenesis in *N. meningitidis* and *N. gonorrhoeae* [9]. Low iron levels are known to exert a bacteriostatic effect over most invading bacteria and the human host exploits this by maintaining low concentrations of free iron within serum and its secretions via iron-binding proteins [10]. Many micro-organisms have evolved to overcome the selective pressure of iron-limited environments and *Neisseria* spp. are known to possess a wide range of mechanisms for its acquisition [11]. It is believed that diversity in iron uptake genes aids colonization of different *Neisseria* spp. within the same niche, where host antibodies are targeted towards a variety of iron acquisition components from different bacterial species [12].

While some strains of *N. meningitidis* have an invasive capacity [13], invasive, disseminated *N. gonorrhoeae* infections are rare. Pathogenic *Neisseria* are most often associated with their own niche environments, with *N. gonorrhoeae* being associated with infections of the mucosa within the genital tract and *N. meningitidis* being associated with the nasopharynx. Despite this being the case, site of infection does not provide an accurate way to identify *Neisseria* spp. [14]. Over the past few decades, cases of *N. meningitidis* isolated from the genital tract mucosa have increased [14, 15] and similarly, cases of *N. gonorrhoeae* isolated from the oropharynx have increased [16, 17]. Indeed, several recent cases of difficult to treat extensively drug resistant (XDR) gonococcal infections, included pharyngeal infection [18, 19]. With regards to the nasopharyngeal and oral cavities in humans, commensal *Neisseria* have been isolated co-colonizing alongside the pathogens *N. meningitidis* and *N. gonorrhoeae *. It is believed that through co-colonization and a natural competence for transformation [20], many of the pathogenic and commensal *Neisseria* now share a significant pool of genetic material with one another and many commensals can be identified as containing most of the virulence genes associated with the pathogens [21].

The pathogenic and commensal *Neisseria* spp. occupy the same niches and as a result of their common ancestry, in combination with sharing of genetic material, possess high levels of similarity across their genomes [22, 23]. An analysis across commensal and pathogenic neisserial genomes carried out by Maiden and Harrison [24] highlighted these similarities, with *N. lactamica* and *N. meningitidis* being found to share a set of core genes that contribute to around 60 % of their overall genome size. In addition, widespread horizontal genetic transfer between human *Neisseria* species has been seen through comparative genomic analysis [12], and pili in commensal *N. elongata* have been demonstrated to be involved in interspecies gene transfer with *N. gonorrhoeae* [25].

Current molecular identification techniques often struggle to distinguish clearly between different bacterial groups in this genus [26], although identification to the level of genus can be achieved through the examination of specific genes or groups of genes. Ribosomal genes are often chosen for identification as they tend to be highly conserved throughout evolution. While phylogenetic grouping using 16S rRNA gene sequences can aid in species identification, issues have arisen when using this method alone due to different species, including *Neisseria* spp., possessing very similar or identical 16S rRNA genes [24]. Analysis of a set of core genes found across all bacteria through Ribosomal Multilocus Sequence Typing (rMLST) has been shown to be a more efficient and rapid method than 16S rRNA typing for bacterial species identification [24].

In this study, four *Neisseria* spp. were isolated from the throats of human volunteers. These were classified using microbiological techniques and their genome sequences were compared against other commensal and pathogenic *Neisseria *. The genomic sequences of these four isolates display a high level of similarity to commensal *Neisseria* spp., although analysis of their genomic sequences has highlighted the presence of sequences classified as virulence genes when present in the pathogens, including capsule, pilus, and LOS, as well as a number of non-pilus adhesins more commonly associated with pathogenic *Neisseria* spp. Specific gene clusters have also been identified that are believed to encode a newly identified CRISPR system, a type VI secretion system, a type IV secretion system different from that in the Gonococcal Genetic Island [27, 28], a hemin transporter, and a haem acquisition and utilization system. The data presented in this study provide further evidence for a diverse and varied gene pool within *Neisseria* spp. and highlight the presence of gene clusters within these isolates with functions still to be explored.

## Methods

### Bacterial isolation

Isolates were obtained by sweeping the back of the throat with a sterile cotton tipped swab and then plating immediately onto GC agar (Oxoid) with Kellogg’s [29] and 5 % Fe(NO_3_)_3_ supplements. Shortly after collection, plates were incubated at 37 °C in a candle tin overnight. KU isolates came from sampling 64 student volunteers on 3 separate occasions in the Spring of 2012. RH isolates came from sampling six student volunteers on four separate occasions from October 2012 to January 2013. From the mixed bacterial cultures obtained, replica plates were taken using a Scienceware replica plater and sterile velveteen. Two replica plates were taken, one to create a freezer stock and one to select individual colonies for isolation. These were incubated overnight at 37 °C in a candle tin. The following day, all growth from one plate was frozen at −80 °C and from the other plate individual colonies were picked onto fresh GC agar with supplements for individual isolation. After overnight growth, cultures were Gram-stained and tested for catalase and oxidase activity. Gram-negative, oxidase-positive, catalase-positive cultures were archived at −80 °C locally and at the National Collection of Industrial Food and Marine Bacteria (NCIMB, Aberdeen).

### Identification of *Neisseria* species

Four suspected *Neisseria* spp. isolates were grown on GC agar plates (Oxoid) with Kellogg’s [29] and 5 % Fe(NO_3_)_3_ supplements at 37 °C with 5 % CO_2_. Control species for India ink staining, *Escherichia coli* and *Bacillus subtilis *, were grown on nutrient agar (Oxoid) and incubated at 37 °C. Blood agar (Oxoid) included 7 % defibrinated horse blood (Oxoid) to test for haemolytic activity. API NH strips (bioMérieux) to identify the species were used according to the manufacturer’s instructions. The Nitrate Reduction Test (Sigma) was performed according to manufacturer’s instructions to identify samples capable of reducing nitrates.

### Genome sequencing

DNA was extracted from the four isolates by scraping the growth from a GC agar plate into 500 µl of GC broth and extracting the DNA using the Puregene Yeast/Bacterial kit (Qiagen). This extracted DNA was dried down and sent to the MicrobesNG service (microbesng.uk) for Illumina sequencing. The sequence data were processed through SPAdes [30] to generate *de novo*-assembled contigs that were automatically annotated using Prokka [31] according to the standard MicrobesNG pipeline. The contigs were also submitted to RAST [32–34] and to the National Center for Biotechnology Information (NCBI) Prokaryotic Genome Annotation Pipeline [35] (software revision 3.3). These automated annotations were compared in Artemis [36] and the NCBI annotation was chosen for use and submission to GenBank: KU1003-01 [37]; KU1003-02 [38]; RH3002v2f [39]; RH3002v2g [40]. Genome sequence data were analysed using rMLST [41], the NCBI 16S blast tool (blastn against the 16S ribosomal RNA sequences database) [42], CRISPRminer [43], CRISPRFinder [44], nucleotide blast [42], clustal Omega [45], and SecRet6 [46].

### Genome sequence analysis

The PubMLST Genome Comparator tool v2.6.2 [47] was used to compare 38 completed *Neisseria* spp. genome sequences and those of KU1003-01, KU1003-02, RH3002v2f, and RH3002v2g. In total, 3023 loci were compared across all genomes, with the following parameters: minimum 70 % identity, minimum 50 % alignment, blastn word size 20, and 80 % core threshold. The resulting distance matrix output was loaded into SplitsTree4 [48] in nexus format to generate a neighbour-joining cladogram. Virulence genes [21] within the genome sequences of the four isolates were identified using progressive Mauve v2.3.1 at default settings [49]. The NCBI-generated annotation files containing the DNA sequence of each of the isolate’s contigs were aligned against *N. subflava* strain M18660 [50], *N. mucosa* strain FDAARGOS_260 [51], *N. flavescens* strain ATCC 13120 [52], *N. cinerea* strain NCTC10294 [53], *N. meningitidis* strains MC58 [54], FAM18 [55], and Z2491 [56], *N. gonorrhoeae* strains FA 1090 [57] and NCCP11945 [58], and *N. lactamica* strain 020–06 [59]. Virulence genes from the pathogen genome sequences were identified in Mauve using the sequence navigator and aligned to reveal the presence of homologous sequences where present in the isolates. Additional investigations used a set of 15 *N *. *subflava* strains [50, 60–73].

## Results and discussion

### Microbiological identification

The four isolates collected from human throat swab cultures were identified as being oxidase- and catalase-positive Gram-negative diplococci. Two isolates, KU1003-01 and KU1003-02, came from the same individual and were collected on the same occasion. The other two isolates, RH3002v2f and RH3002v2g, also came from a single volunteer and were collected on the same occasion. No other Gram-negative oxidase- and catalase-positive isolates were identified. The concurrent collection of these isolates supports co-colonization of this human niche with more than one neisserial species at a time, as proposed by Yazdankhah and Caugant [74].

Isolate KU1003-01 presented as large, smooth, round, and moist colonies on GC agar producing a yellowish pigment and having a glistening surface. Isolate KU1003-02 presented as medium, round, and slightly granular colonies on GC agar producing a yellowish pigment and having a rough surface. Isolate RH3002v2f presented as small, round, unpigmented colonies with a glistening surface. Isolate RH3002v2g presented as medium, round, and smooth pigmented colonies with a glistening surface.

None of the isolates were determined to be haemolytic; a lack of haemolysis on blood agar indicated that the isolates did not belong to the haemolytic species *N. animaloris* [75]. Nitrate reduction testing for all four isolates gave negative results, indicating the isolates could not be *N. mucosa *, *N. oralis *, *N. canis *, *N. shayeganii *, *N. wadisworthii*, or *N. elongata* subspecies *nitroreducens* [76]. All four isolates grew at 35 °C, indicating that they were not *N. gonorrhoeae *, according to the API NH growth criteria [76]. A control *N. gonorrhoeae* strain NCCP11945 culture did not grow under these conditions. API NH results indicated that the best identification of three of the isolates was as *Neisseria* spp. (Table 1). Isolate RH3002v2f is most likely to be *Neisseria cinerea,* based on the result of the API NH test, growth at 35 °C, and its translucent and glistening appearance [77].

**Table 1.** Isolate identification using API NH, rMLST, and 16S blast analysis

Isolate	API NH results	rMLST	Top 16S blast hit (NCBI)
KU1003-01	* Neisseria* spp.	*N. flavescens, N. mucosa, N. subflava*	* N. perflava* (99 %, E value 0.0)
KU1003-02	* Neisseria* spp.	* N. mucosa *	* N. perflava* (99 %, E value 0.0); *N. cinerea* (99%, E value 0.0))
RH3002v2f	* Neisseria cinerea *	* N. cinerea *	* N. cinerea* (99 %, E value 0.0); *N. meningitidis* (99 %, E value 0.0)
RH3002v2g	* Neisseria* spp.	*N. flavescens, N. mucosa, N. subflava*	* N. perflava* (99 %, E value 0.0)

### Genome sequencing and assembly

Genome sequencing and assembly for all four isolates was carried out by MicrobesNG. Illumina Mi-seq short reads were *de novo* assembled into contiguous sequences using SPades. The assembly of sequence reads for KU1003-01 generated 58 contigs with a total genome size of 2 345 197 bp [37]. Isolate KU1003-02 assembled into the greatest number of contigs at 73. Despite this, the overall genome size for this isolate is on a par with the size of its co-isolate at 2 303 261 bp [38]. Isolate RH3002v2f contains the smallest of the three genomes at 1 953 373 bp, which assembled in 26 contigs [39]. By comparison, its co-isolate RH3002v2g has a genome size of 2 193 423 bp across 42 contigs [40].

### Genome sequence-based identification

*Neisseria* spp. share common ancestry, occupy the same niche, and are able to share genetic material. As a direct result, *Neisseria* spp. display high levels of similarity across their genomes [22, 23]. Current molecular identification techniques often struggle to distinguish clearly between different bacterial groups [26] and difficulties can arise when assigning *Neisseria* to particular species groups due to their genetic similarity [26].

Analysis of the genome sequence data through rMLST and 16S blast suggested a range of *Neisseria* spp. for the four isolates (Table 1). While 16S rRNA analysis was able to classify the isolates used in this study as *Neisseria* spp., the results of the rMLST indicated varying results with regard to their identification as one particular species. The result of rMLST for isolate KU1003-01 indicated that it was either *N. flavescens *, *N. mucosa *, or *N. subflava *, but the top 16S blast hit suggested *N. perflava *, which is a biovar of *N. subflava* (Table 1). For KU1003-02, rMLST indicated that it was *N. mucosa *, whilst the 16S disagreed, suggesting *N. perflava* or *N. cinerea* (Table 1). Analysis of RH3002v2f through rMLST indicated that this isolate was *N. cinerea* and 16S homology also suggested *N. cinerea* or *N. meningitidis *. Therefore, the sequence-based results support the laboratory results, suggesting that this isolate is *N. cinerea* (Table 1). The fourth isolate, RH3002v2g, produced the same rMLST and 16S rRNA blast results as KU1003-01, suggesting that it was either *N. flavescens *, *N. mucosa *, or *N. subflava *, and *N. perflava* (* N. subflava* biovar *perflava*), respectively (Table 1).

### Signatures of DNA Uptake Sequences (DUSs) support species assignments

In the pathogenic *Neisseria* spp., the spread and increased levels of antibiotic resistance, as well as the evolution of pathogenesis, are as a direct result of their ability to take up and transform DNA from their environment [23, 74, 78, 79].

To identify and locate DUSs, the four isolate genome sequences were subjected to frequent character analyses as described by Davidsen *et al*. [80]. Within the KU1003-01 genome sequence data, 2009 copies of DUS variant 1 (DUSvar1) [81] were identified. DUSvar1 is also referred to as AG-DUS [82]. Within the KU1003-02 genome sequence data, 2393 copies of DUSvar1/AG-DUS were identified and within RH3002 v2g, 1957 copies of DUSvar1/AG-DUS were identified (Table 2). DUSvar1/AG-DUS are most often associated with *N. subflava *, *N. flavescens,* and *N. elongata* [81, 82]. The dialects in neisserial DUS signatures are known to vary in a species-specific manner [12, 81, 82]. There are 2024 copies of DUSvar1/AG-DUS in *N. subflava* strain M18660, for example [Data Citation 5]. A significantly lower number of DUS variant 2 (DUSvar2), also known as AG-mucDUS, associated with *N. mucosa* and *N. sicca* [81, 82], were identified in these three isolates (Table 2). These data support the assignment of isolates KU1003-01, KU1003-02, and RH3002v2g to *N. subflava* biovar *perflava*. The classical DUS described by Berry *et al*. [81] and designated AT-DUS by Frye *et al*. [82] is associated with *N. meningitidis *, *N. gonorrhoeae *, *N. lactamica,* and *N. cinerea *. This was the most frequent DUS identified within isolate RH3002v2f, at 1158 copies, and this result is consistent with the API NH and rMLST data for this isolate belonging to the species *N. cinerea *.

**Table 2.** The type and number of DUSs within sequenced isolates and their best match genome sequences for comparison

	Classical DUS [81]/AT-DUS [82]	DUSvar1 [81]/AG-DUS [82]	DUSvar2 [81]/AG-mucDUS [82]
	ATGCCGTCTGAA	AGGCCGTCTGAA	AGGTCGTCTGAA
**KU1003-01**	**140**	**2009**	**99**
**KU1003-02**	**172**	**2393**	**107**
**RH3002 vg**	**137**	**1909**	**83**
*** N. subflava* M18660**	**147**	**2024**	**98**
**RH3002 vf**	**1158**	**202**	**74**
*** N. cinerea* NCTC10294**	**1198**	**212**	**80**

### Comparative genome sequence analysis

**Fig. 1.**
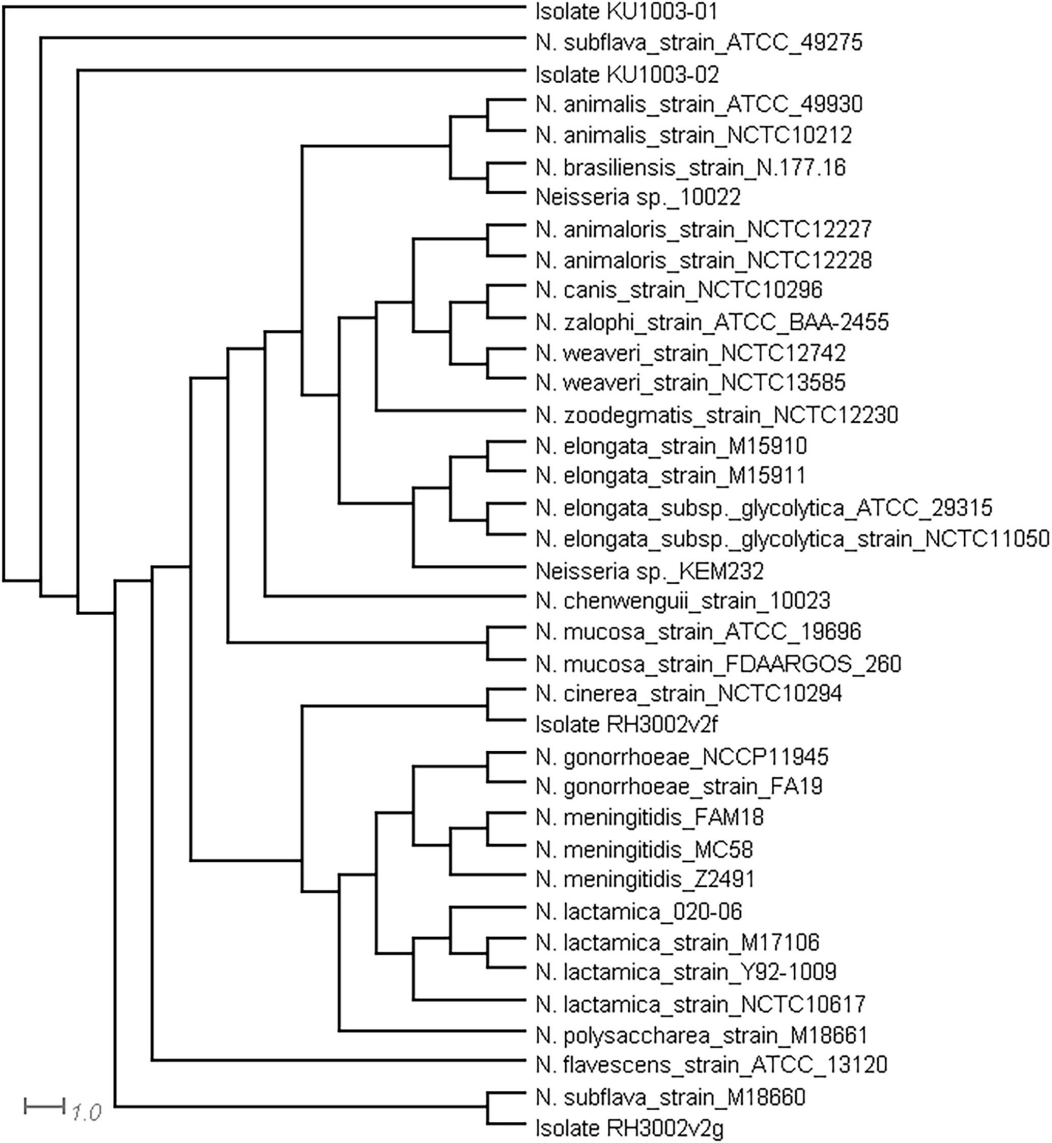
Neighbour-joining cladogram tree of the four *Neisseria* spp. isolates sequenced. Generated using the PubMLST Genome Comparator tool at Neisseria.org [47] and SplitsTree4 [[48]] with the complete genome sequences of 38 *Neisseria* spp. and the sequence data from isolates KU1003-01, KU1003-02, RH3002v2f, and RH3002v2g.

To further support the species assignments, phylogenetic analysis was conducted using the genomic sequences of the isolates, compared to complete genome sequence data from 38 *Neisseria* spp. in the PubMLST database on Neisseria.org [ 47]. The output was visualized using SplitsTree4 [48], which showed that isolates KU1003-01 and KU1003-02 clustered with *N. subflava* strain ATCC 49275, RH3002v2g with *N. subflava* strain M18660, and RH3002v2f with *N. cinerea* strain NCTC10294 (Fig. 1). This adds another level of support to our species assignments for these isolates.

**Fig. 2.**
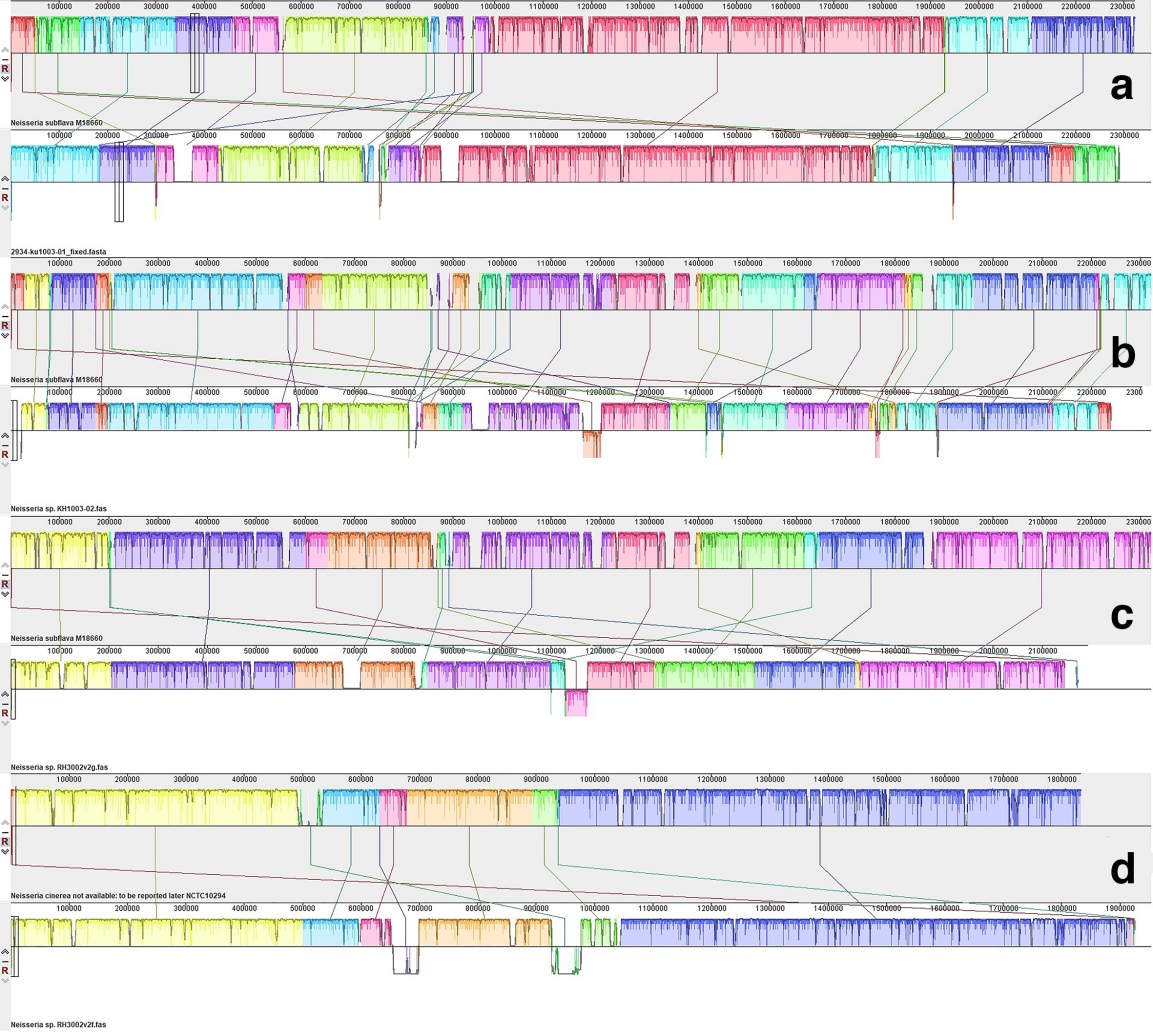
Whole-genome progressive Mauve pairwise alignments of ordered contigs against complete genome sequences that are similar to those of the isolates. Blocks that are the same colour between the aligned pairs show regions of genome sequence data that share homology. Three of the isolates aligned well to the complete genome sequence for *N. subflava* strain M18660, as shown in pairs A (KU1003-01 and *N. subflava* strain M18660), B (KU1003-02 and *N. subflava* strain M18660), and C (RH3002v3g and *N. subflava* strain M18660). One isolate, RH3002v2f, aligned well with *N. cinerea* NCTC10294, as in pair D.

The genome sequences and annotations for each of the four isolates, KU1003-01, KU1003-02, RH3002v2f, and RH3002v2g, were imported into Mauve and compared against commensal *Neisseria* spp. reference sequences to identify and align homologous regions. To facilitate comparative analyses between the genomes, the contigs for each isolate were reordered against a reference genome believed to be most similar to the isolate using the Mauve contig mover (MCM) tool (Fig. 2). KU1003-01, KU1003-02, and RH3002 v2g aligned closely to the completed genome for *N. subflava* M18660 [50] rather than *N. mucosa* strain FDAARGOS_260 [51] or *N. flavescens* strain ATCC 13120 [52]. RH3002v2f aligned closely to the completed genome for *N. cinerea* NCTC 10294 [53]. These alignments strongly support KU1003-01, KU1003-02, and RH3002 v2g being *N. subflava* biovar *perflava*, and RH3002 v2f being *N. cinerea *, agreeing with the laboratory and other genetic analyses. Comparisons between the co-isolated genomes also indicated that despite originating from the same host, KU1003-01 and KU1003-02, identified as *N. subflava* biovar *perflava*, came from two distinct lineages.

Genome sequence comparison is considered to be an effective tool for identifying putative virulence genes [83] and regions of difference between species. Mauve alignments against *N. meningitidis* strains MC58 [54], FAM18 [55], and Z2491 [56], *N. gonorrhoeae* strains FA 1090 [57] and NCCP11945 [58], and *N. lactamica* strain 020–06 [59] were used to assess similarities with other *Neisseria* spp. reference sequences, including the pathogens. These comparisons revealed regions of similarity for further investigation (Table 3), as well as regions of difference that were also investigated (Table 4), some of which were unique to a particular isolate.

**Table 3.** A comparison of the presence of virulence genes across the isolates

Virulence factors	Related genes	* Neisseria* spp. KU1003-01	* Neisseria* spp. KU1003-02	* Neisseria* spp. RH3002v2f	* Neisseria* spp. RH3002v2g
**Adherence**					
Adhesion and penetration protein	*app/hap*	np	np	np	np
LOS sialylation	*lst*	np	np	np	np
LOS synthesis	*lgtA*	np	np	BBW69_00125	np
	*lgtB*	np	np	BBW69_00130	np
	*lgtC*	np	np	np	np
	*lgtD*	np	np	np	np
	*lgtE*	np	np	BBW69_00135	np
	*lgtF*	np	BBP28_02055	BBW69_04775	np
	*lgtG*	np	np	BBW69_09635, BBW69_09640‡	np
	*rfaK*	np	BBP28_02060	BBW69_04770	np
	*rfaF*	BBW80_07160	BBP28_02020	BBW69_06390	BBP27_02875
	*kdtA/waaA*	BBW80_02890	BBP28_00140	BBW69_08680	BBP27_08930
	*rfaC*	BBW80_03315	BBP28_01425	BBW69_08770	BBP27_00620
	rfaD*	BBW80_06690	BBP28_07150	BBW69_01995	BBP27_00270
	*rfaE* *	BBW80_06685	BBP28_07145	BBW69_01990	BBP27_00265
	*lpxA* *	BBW80_02940	BBP28_00185	BBW69_08210	BBP27_04090
	*lpxB* *	BBW80_02935	BBP28_00180	BBW69_08320	BBP27_04085
	*lpxD* *	BBW80_02950	BBP28_00195	BBW69_08220	BBP27_04100
	*pgm* *	BBW80_07200	BBP28_01980	BBW69_01850	BBP27_02915
	*misR* *	BBW80_10070	BBP28_05700	BBW69_00920	BBP27_05185
	*misS* *	BBW80_10075	BBP28_05695	BBW69_00915	BBP27_05190
	*fabZ* *	BBW80_02945	BBP28_00190	BBW69_08215	BBP27_04095
	*kdsA*	BBW80_07275	BBP28_00980	BBW69_02905	BBP27_03005
	*kdsB*	BBW80_03730	BBP28_07120	BBW69_01220	BBP27_00240
* Neisseria* adhesion A NadA	*nadA*	BBW80_10475	†	np	BBP27_04320
Type IV pili	*pilC1*	BBW80_01810	BBP28_10510	BBW69_04085	BBP27_07165
	*pilC2*	np	np	BBW69_04075	np
	*pilE*	BBW80_10385	BBP28_05385	BBW69_08575	BBP27_05490
	*pilS*	BBW80_10390	BBP28_05390	np	BBP27_05495
	*pilD*	BBW80_01845	BBP28_10475	BBW69_05880	BBP27_07235
	*pilF*	BBW80_01835	BBP28_10485	BBW69_05895	BBP27_07225
	*pilG*	BBW80_01840	BBP28_10480	BBW69_05875	BBP27_07230
	*pilX*	BBW80_07320	BBP28_01030	BBW69_07215	BBP27_03050
	*pilW*	BBW80_07325	BBP28_01035	BBW69_07210	BBP27_03055
	*pilM*	BBW80_06120	BBP28_04890	BBW69_05370	BBP27_05990
	*pilN*	BBW80_06125	BBP28_04895	BBW69_05375	BBP27_05985
	*pilO*	BBW80_06130	BBP28_04900	BBW69_05380	BBP27_05980
	*pilP*	BBW80_06135	BBP28_04905	BBW69_05385	BBP27_05975
	*pilQ*	BBW80_06140	BBP28_04910	BBW69_05390	BBP27_05970
	*pilT*	BBW80_03545	BBP28_03915	BBW69_08555	BBP27_04600
	*pilT2*	BBW80_03550	BBW80_03550	BBW69_08560	BBP27_04605
	*pilU*	BBW80_09540	BBP28_08495	BBW69_01715	BBP27_08040
	*pilH*	BBW80_07335	BBP28_01045	BBW69_07200	BBP27_03065
	*pilV*	BBW80_07330	BBP28_01040	BBW69_07205	BBP27_03060
	*pilZ*	BBW80_03840	BBP28_07015	BBW69_01725	BBP27_00140
Pilus-associated genes	*pglA* *	np	np	np	np
	*pglB* *	np	np	BBW69_05440	np
	pglB2 *	BBW80_06065	BBP28_04835	np	BBP27_09005
	pglB2b *	BBW80_06060	BBP28_04830	np	BBP27_09000
	*pglC* *	BBW80_06045	BBP28_04815	BBW69_05445	BBP27_09025
	*pglD* *	BBW80_06040	BBP28_04810	BBW69_05450	BBP27_09030
	*pglE* *	np	np	np	np
	pglF*	BBW80_06085	BBP28_04855	BBW69_05425	BBP27_08985
	*pglG* *	BBW80_06075	BBP28_04845	BBW69_05430	BBP27_08995
	*pglH* *	BBW80_06070	BBP28_04840	BBW69_05435	BBP27_09000
**Efflux pump systems**					
FarAB	*farA*	BBW80_10400	BBP28_04660	BBW69_05945	BBP27_04245
	*farB*	BBW80_10395	BBP28_04655	BBW69_05940	BBP27_04240
MtrCDE	*mtrC*	BBW80_00055	BBP28_10630	BBW69_04715	BBP27_01915
	*mtrD*	BBW80_00060	BBP28_10625	BBW69_04720	BBP27_01910
	*mtrE*	BBW80_00065	BBP28_10620	BBW69_04725	BBP27_01905
	mtrR *	BBW80_00050	BBP28_10635	BBW69_04710	BBP27_01920
**Immune evasion**					
Capsule	*ctrA*	BBW80_02740	BBP28_10005	np	BBP27_08790
	*ctrB*	BBW80_02735	BBP28_10000	np	BBP27_08785
	*ctrC*	BBW80_02730	BBP28_09995	np	BBP27_08780
	*ctrD*	BBW80_02725	BBP28_09990	np	BBP27_08775
	*lipA*	BBW80_08850	BBP28_09040	np	BBP27_03705
	*lipB*	BBW80_08845	BBP28_09045	np	BBP27_03710
	*siaA / synA*	np	np	np	np
	*siaB/synB*	np	np	np	np
	*siaC/synC*	np	np	np	np
	*siaD/synD*	np	np	np	np
	*synE*	np	np	np	np
	*mynA / sacA*	np	np	np	np
	*mynB/sacB*	np	np	np	np
	*mynC/sacC*	np	np	np	np
	*mynD/sacD*	np	np	np	np
**Immune modulators**					
Factor H binding	*fHbp*	BBW80_06745	np	BBW69_06045	np
Neisserial surface protein A	*nspA*	np	np	BBW69_01155	np
**Invasion**					
Class 5 outer protein	*opc*	np	np	np	np
Opacity protein	*opa*	np	np	BBW69_01160	np
PorA	*porA*	np	np	np	np
PorB	*porB*	BBW80_10465	BBP28_04620	BBW69_04225	BBP27_04310
Other surface proteins	*omph* *	BBW80_02955	BBP28_00200	BBW69_08225	BBP27_04105
	*omp*85 *	BBW80_02960	BBP28_00205	BBW69_08230	BBP27_04110
Heparin-binding antigen NHBA	*nhba*	np	np	np	np
**Iron uptake systems**					
ABC transporter	*fbpA*	np	np	BBW69_01085	np
	*fbpB*	np	np	BBW69_01080	np
	*fbpC*	np	np	BBW69_01075	np
Ferric enterobactin transport protein A/ferric-repressed protein B	*fetA / frpB*	BBW80_05725	BBP28_00435	BBW69_09195	BBP27_09310
Haemoglobin receptor	*hmbR*	BBW80_00620	BBP28_10830	np	BBP27_01375
Haem uptake	*hpuA*	np	np	np	np
	*hpuB*	np	np	BBW69_08435	np
Lactoferrin-binding protein	*lbpA*	np	np	BBW69_01315	np
	*lbpB*	np	np	BBW69_01310	np
Ton system	*tonB*	BBW80_02960	BBP28_00205	BBW69_08230	BBP27_04110
	*exbB*	BBW80_01265	BBP28_01625	BBW69_04660	BBP27_06930
	*exbD*	BBW80_01260	BBP28_01620	BBW69_04665	BBP27_06925
Transferrin-binding protein	*tbpA*	BBW80_03495	BBP28_03865	BBW69_08600	BBP27_04660
	*tbpB*	BBW80_04640	np	np	np
Other iron acquisition genes	*bcp* *	BBW80_04540	BBP28_06595	BBW69_01630	BBP27_09650
	*bfrA* *	BBW80_08395	BBP28_02745	BBW69_03245	BBP27_06475
	*bfrB* *	BBW80_08400	BBP28_02740	BBW69_03240	BBP27_06480
	*hemH* *	BBW80_04410	BBP28_06720	BBW69_01460	BBP27_09780
**Protease**					
IgA protease	*Iga*	np	np	np	np
**Stress proteins**					
Catalase	*katA*	BBW80_05740	BBP28_00450	BBW69_04130	BBP27_09245
Manganese transport system	*mntA*	BBW80_00475	BBP28_10980	BBW69_00885	BBP27_01220
	*mntB*	BBW80_00480	BBP28_10975	BBW69_00880	BBP27_01225
	*mntC*	BBW80_00485	BBP28_10970	BBW69_00875	BBP27_01230
Methionine sulphoxide reductase	*msrA / msrB* (*pilB*)	BBW80_03270	BBP28_01380	BBW69_08590	BBP27_00665
Recombinational repair protein	*recN*	BBW80_04510	BBP28_06620	BBW69_01565	BBP27_09675
**Toxin**					
RTX toxin	*frpA*	np	np	np	np
	*frpC*	np	np	np	np
Two comp reg sys	*basR*	BBW80_06160	BBP28_04930	BBW69_05315	BBP27_05950
	*basS*	BBW80_06155	BBP28_04925	BBW69_05310	BBP27_05955
Cell separation	*nlpD*	BBW80_08360	BBP28_02780	BBW69_06215	BBP27_06440
Nitric oxide reductase	*norB*	BBW80_04740	BBP28_06390	BBW69_06810	BBP27_01665

*Previously reported as pathogen-specific.

†*nadA* homologue of *Yersinia yadA* was predicted by RAST as a partial gene at the end of 2 contigs

‡Two adjacent CDSs align.

np, not present.

**Table 4.** Specific regions of difference identified across the four isolates

Isolate	Annotated CDSs	Annotated functions
KU1003-01	BBW80_02625 to BBW80_02655	Type VI secretion system
KU1003-01	BBW80_00155 to BBW80_00225	CRISPR system
KU1003-02	BBP28_10015 to BBP25_10115	Type IV secretion system
RH3002v2f	BBW69_00525 to BBW69_00530	Two-partner putative haemolysin secretion system
RH3002v2g	BBP27_06840 to BBP27_06885	TonB-dependent haem acquisition and utilization

### Virulence genes present in the commensal isolates

Many virulence factors in the pathogenic *Neisseria* have been identified to date and this has been the focus of a great deal of research due to their importance in public health [6, 84–86]. Within the regions of similarity identified in comparative genome analysis, the presence of a set of 117 virulence genes was assessed in each of the 4 isolates by comparison against the pathogen genome sequences in Mauve. Of the 117 virulence genes investigated, 94 are present in 1 or more of the isolate genome sequences (Table 3). There is some strain-to-strain variation in the presence of homologous sequences, but for some they are present across all of the isolate genomes. For example, there are homologous sequences for the majority of the neisserial type IV pilus genes (Table 3). As has been reported previously [21], it is likely that some of what have been identified as ‘virulence genes’ require reclassification in light of their presence in the non-pathogenic *Neisseria* spp. genomes and the role for some of these in niche survival, rather than pathogenicity. Virulence genes shared between the commensal and pathogenic species include those involved in host adhesion, invasion, and immune response evasion, with adhesion being critical for successful host colonization [87, 88]. Coding sequences (CDSs) for two efflux pump systems, MtrCDE and FarAB, were identified; these have been investigated in *N. gonorrhoeae* for their roles in survival against mucosal surface fatty acids and bile salts [89–91].

### Possession of type IV pili sequences

Initial binding to host cells, which is important for colonization and microcolony formation, is achieved through the actions of the type IV pili in the pathogenic *Neisseria* spp. [92]. Commensal *Neisseria* spp. are also known to be able to produce these structures; electron microscopy confirmed their presence decades ago within *N. perflava* and *N. subflava* [93], and the pili of some commensal *Neisseria* spp. have been demonstrated to have the capacity to adhere to human epithelial cells [94].

The majority of the neisserial pilus genes were identified in all four isolates (Table 3), including those necessary for its biogenesis. Similar to the observations of Marri *et al*. [12], the *pilC* sequences identified in the four isolates appear to be orthologues of those found in the pathogens. In the pathogens, PilC is known to be involved in pilus-mediated adhesion as well as pilus biogenesis and is normally present in two copies [95]. A single CDS with homology to *pilC* was identified in the assembled genomic data for isolates KU1003-01, KU1003-02, and RH3002v2g, while isolate RH3002v2f has two copies. Although the function of the commensal *pilC* orthologue has not yet been elucidated [12], its presence as an intact CDS within the isolates indicates that these commensals likely have the capacity to produce pili.

All four isolates possess CDSs with homology to the major pilus structural subunit PilE, which associates to form the pilus fibre [96]. In the pathogens, *pilE* is also responsible for mediating antigenic variation, achieved through recombination with silent *pilS* pilin sequences [97]. A single copy of *pilE* was identified in the isolates and a single copy of *pilS* could be found in the assembled genomic data. In general, the commensals are thought to only have 2–5 copies of *pilS*, but in the pathogenic *Neisseria,* up to 19 copies have previously been reported [12].

Pathogen-specific pilus gene sequences were also identified in all four isolates, including *pglB*, *pglC*, *pglD*, *pglF*, *pglG,* and *pglH*. These genes are believed to be necessary for complement-mediated lysis resistance in meningococci through pilin glycosylation [98]. Within the three isolates, RH3002v2f has CDSs for *pglB*, *pglC*, *pglD*, *pglF*, *pglG,* and *pglH,* at one locus. In KU1003-01, KU1003-02, and RH3002v2g, two alleles of *pglB* were identified (*pglB2* and *pglB2b*). Similar to the observations made by Kahler *et al*. in *N. meningitidis* [98], a CDS was found to be inserted between *pglB*2 and *pglC* in these three isolates.

### Presence of previously unreported capsule loci sequences

**Fig. 3.**
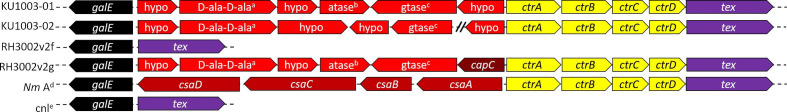
The capsule loci of the four commensal isolates were each different and distinct. For KU1003-01 (line 1), KU1003-02 (line 2) and RH3002v2g (line 4) there are *ctr* capsule transport genes (yellow) and potentially capsular serogroup defining genes (red) between *galE* and *tex*, as in *N. meningitidis *. An example of a serogroup A meningococcal capsular locus is shown (*Nm* A line 5). The sequence for KU1003-02 is across two contigs, indicated by \\. RH3002v2f (line 3) does not have *ctr* or potential capsule biosynthesis genes, rather having a similar locus to the capsule null loci (cnl) found in some meningococci and *N. lactamica* strain 020–06. ^a^ d-ala-d-ala ligase; ^b^ acetyltransferase; ^c^ glycosyl transferase. ^d, e^ are from [100], as is the colour scheme of regions defined previously: D (black); A (red); C (yellow); and E (purple).

This investigation is the first to identify within the non-pathogenic *Neisseria *, capsule gene loci sequences that have not previously been characterized (Fig. 3). Analysis of the genome sequence data for KU1003-01, KU1003-02, and RH3002v2g (Table 3) indicates that these isolates contain a number of capsule CDSs homologous to those found in *N. meningitidis* (Fig. 3). While no capsule CDSs were identified within RH3002v2f, this isolate was found to contain sequences with similarities to those found in *N. lactamica* strain 020–06 at the syntenic genomic region (Fig. 3) [51, 99].

The structure of the capsule locus is well characterized and conserved in *N. meningitidis* [100, 101] and phylogenetic analysis of *Neisseria* spp. capsule genes carried out by Clemence *et al*. [7] highlighted that *N. subflava* was the closest encapsulated relative of *N. meningitidis *. Putative capsule genes with synthesis, transport, and translocation functions have previously been reported in the non-pathogenic *Neisseria* spp. [7] and similar to the previous findings for *N. subflava,* these putative regions were found to be contiguous in isolates KU1003-01 and RH3002v2g and one contig break in the locus occurs in KU1003-02 (Fig. 3).

While the capsule loci of the four commensal isolates were each different and distinct from those in the pathogenic *N. meningitidis *, capsular synthesis and potential serogroup-defining genes were identified within isolates KU1003-01, KU1003-02, and RH3002v2g (Fig. 3). Similar to the organization of the capsule loci in *N. meningitidis* serogroup A, the genes involved in capsular synthesis and serogroup definition were identified in isolates KU1003-01, KU1003-02, and RH3002v2g, flanked on both sides by genes involved in capsule transport and capsule translocation.

Homology of capsule loci shared between *N. meningitidis* and other commensal genomes has indicated that some non-pathogens could represent a reservoir for capsule switching [6, 102, 103]. The acquisition of the capsular genes by *N. meningitidis* from the non-pathogenic *Neisseria* spp. evolutionarily [7, 101] and the recent discovery of meningococcal capsule genes in the newly described putatively named *Neisseria brasiliensis* [103] support the capacity for interspecies transfer of capsular genes between the non-pathogens and *N. meningitidis *. Although new meningococcal serogroups have not been identified in *N. meningitidis,* capsule switching could provide a means for circumventing the serogroup specific vaccines directed against it. This would, however, be dependent on the pathogen horizontally acquiring capsular gene sequences from a co-colonizing commensal species in combination with retaining its pathogenicity. Capsule switching in combination with the acquisition of alleles for other vaccine targets, such as sequences for fHbp and NadA (Table 3), that are divergent from the alleles represented in the Bexsero vaccine [104, 105], could provide routes for vaccine escape via horizontal gene transfer (HGT) from these commensal genomes. Vaccine targets NHBA and PorA were not found in these isolate genome sequences (Table 3).

The capsule of some *N. meningitidis* serotypes are considered to be a major pathogenicity factor and its anti-phagocytic properties are essential for growth in the host’s bloodstream [106]. Despite the defined role of the *N. meningitidis* capsule in virulence*,* its ecological role is not as well defined, as non-encapsulated *N. meningitidis* strains are able to grow and survive within the human nasopharynx as well as encapsulated strains, likely better [6, 74, 101]. Further research is needed to determine the role and nature of the potential capsules in the commensal isolates.

### Presence of vaccine antigen target sequences that demonstrate diversity in the gene pool of the genus

An adhesin/invasin similar to *Neisseria* adhesin A (NadA) is present in the genome sequences of isolates KU1003-01 and RH3002v2g, with a *yadA* homologue in KU1003-02 (Table 3). Submission of these CDSs to Bexsero Antigen Sequence Typing through PUBMLST (https://pubmlst.org/neisseria/NadA/) indicates that the closest match for the protein sequences is *N. meningitidis* NadA-1, with E values of 2e-08 and 8e-10, respectively. Therefore, although present, the NadA that would be expressed in these *N. subflava* biovar *perflava* isolates is predicted to be distinct from those in the Bexsero meningococcal vaccine. In *N. meningitidis *, *nadA* assembles at the cell surface and promotes tight adherence followed by invasion of host epithelial cells [107]. Previous investigations into *N. lactamica* found no CDSs with homology to *nadA* or Bexsero target fHbp [108], both of which are found in these commensal isolates (Table 3). This analysis demonstrates additional reservoirs for antigenic variant alleles of NadA and Fhbp not represented in the vaccine that may allow *N. meningitidis* to escape vaccine-mediated control via HGT from commensal *Neisseria* spp. In concert with the potential role for commensal capsule loci to provide genetic material for capsule switching [6, 101], the scope for evolution of this pathogen, and also for pharyngeal *N. gonorrhoeae *, through their natural competence preference for the neisserial DNA uptake sequence [78, 81, 82] likely contributes to genome plasticity [109].

### Regions of difference within the commensal isolates contain previously explored sequences

Mauve alignments also revealed previously unexplored regions of difference between the four isolate genome sequences. These included both regions that were not present in the other sequenced commensal isolates and regions that were not present in the pathogens and *N. lactamica* reference strains against which they were compared. Five key regions were identified (Table 4), which were investigated in further detail. Similar to the presence of virulence genes within commensal *Neisseria* spp., some of these systems are more often associated with pathogens.

### Presence of a different Type IV Secretion System

Horizontal exchange of genetic material in *N. gonorrhoeae* is facilitated through a multicomponent Type IV Secretion System (T4SS), encoded within the Gonococcal Genetic Island (GGI), present in around 80 % of gonococcal strains [27]. This GGI T4SS has also been identified in some *N. meningitidis* [28], with different capsular serogroup strains containing both complete and partial versions of the GGI [110]. In *N. meningitidis *, however, this system does not secrete DNA, although its GGI T4SS may be responsible for secreting other effectors [111]. T4SSs in the non-pathogenic *Neisseria* spp. have not previously been characterized within any of the commensal *Neisseria* spp. [27].

A T4SS system similar to VirB/D in *Agrobacterium tumefaciens* (i.e. VirB9 1e-42 at 93 % coverage and VirB11 7e-55 at 90 % coverage) was identified within the genome sequence of isolate KU1003-02 (Table 4). blastp revealed no similarity between the T4SS components found in isolate KU1003-02 and the *N. gonorrhoeae* GGI T4SS. blastn of the individual VirB/D components could not identify the same system in the other three isolates, or in any *N. gonorrhoeae *, *N. meningitidis *, or the vast majority of other *Neisseria* spp. in the sequence databases. Orthologues were detected in two of the investigated *N. subflava* biovar *perflava* genomes [67, 72].

The T4SS in KU1003-02 contains CDSs potentially encoding 11 out of the 12 core proteins (VirB1–VirB11 and VirD4) normally associated with this type of secretion system [112]. The Browse by Replicon function within the SecReT4 database identified organizational synteny between the KU1003-02 T4SS and the VirB/D system in *Taylorella equigenitalis* [113]. A CDS with homology to *virB*7 could not be identified within KU1003-02, although a hypothetical protein was predicted at the syntenic location.

Although it is clear that this T4SS has an independent origin from that within the GGI in the pathogenic *Neisseria* spp., the role of the T4SS in isolate KU1003-02 is currently unclear from the data, and therefore further investigation is required.

### Presence of CRISPR systems

Individual species need mechanisms for maintaining their genetic identity [114] and preventing the loss of advantageous genes necessary for their survival and proliferation. CRISPR systems have been proposed as one mechanism by which this is possible and genome size as well as the ability to acquire new genes have been shown to differ between strains that either possess or lack CRISPR systems [115]. CRISPR systems are present in around 40 % of sequenced bacterial genomes, including *Neisseria* spp. [116], and provide acquired, heritable immunity against the acquisition and genomic incorporation of DNA from invading plasmids and bacteriophages [117]. CRISPR use enzymes to degrade foreign DNA that is either identical or very closely related to previously acquired short DNA sequences [117, 118].

A region of difference was identified in the genome sequence of isolate KU1003-01, annotated as a CRISPR locus (Table 4). To investigate CRISPR sequences in the isolates in more depth, the genome sequences for all four isolates were uploaded to CRISPRminer (http://www.microbiome-bigdata.com/CRISPRminer) [43] and CRISPRfinder (https://crispr.i2bc.paris-saclay.fr/Server/) [44]. Isolate KU1003-01 was found to have three confirmed CRISPR loci with a total of 109 spacers (Table 5) according to CRISPRfinder. CRISPRminer identified at least one of the spacers in isolate KU1003-01 as being complementary to *Escherichia* phage HY01, with another being identified as self-targeting. The remainder of the spacers for KU1003-01 did not yield any blast hits through the NCBI database. KU1003-02 was found to have two confirmed CRISPR loci with a total of 111 spacers according to CRISPRfinder (Table 5). One spacer was identified as being self-targeting and no phage complement spacers were identified within isolate KU1003-02 according to CRISPRminer. Isolates RH3002v2f and RH3002 v2g were not identified as having CRISPR using these tools, although Cas proteins were identified within their genomic sequences. Lack of bacteriophage hits and inability to identify CRISPR in genome sequences containing Cas protein homologues suggest that these tools may not be able to recognize the diverse nature of the neisserial CRISPR and bacteriophages.

**Table 5.** A comparison of genome size and GC content (%) as well as the number of CRISPR loci, spacer number and type across the four isolates

Strain/isolate	Genome size (bp)	% G+C	CRISPR loci	Self-targeting	Phage spacer	Spacer no.
KU1003-01	2 345 197	49.00	3	1	1	109
KU1003-02	2 303 261	49.40	2	1	0	111
RH3002v2f	1 953 373	50.60	0	0	0	0
RH3002v2g	2 193 423	49.60	0	0	0	0

While CRISPRs are known to provide adaptive immunity through the incorporation of spacers from invading plasmids and bacteriophages, analysis of these systems across a large number of archaea and bacteria genomes also identified CRISPR spacers that were derived from chromosomal DNA [119]. These ‘self-targeting’ spacers are complementary to non-CRISPR genomic regions within the species in which they were found. While it is currently thought that the most likely outcome for cells containing a complementary self-targeting spacer is death through host autoimmune suicide [117, 119], these spacers may also play a role in maintaining host genome integrity [118–120]. It may be that the CRISPR systems identified in isolates KU1003-01 and KU1003-02 have a role in maintaining their genome integrity during co-colonization with other *Neisseria* spp.

### First identification of a Type VI Secretion System in the *Neisseria* spp

One region of difference found in KU1003-01 included annotated CDSs for a Type VI Secretion System (T6SS) [121, 122], encoding proteins such as EvpB (TssB), Hcp (TssD), ImpG (TssF), VgrG, and PAAR (Table 6). This region was investigated further, identifying a full complement of T6SS sequences across contigs, suggesting that KU1003-01 is able to make a functional T6SS. This is the first report of the potential for Type VI Secretion in this genus.

**Table 6.** CDSs with homology to T6SS components present in three of the isolates

Name	KU1003-01	KU1003-02	RH3002v2g	T6SS component
TssA	BBW80_02650	BBP28_06040	BBP27_04500	Cytosolic protein
TssB/EvpB	BBW80_01505	BBP28_06045	BBP27_04505	Contractile sheath – small subunit
TssC	BBW80_01510	BBP28_06050	BBP27_04510	Contractile sheath – large subunit
TssD/Hcp	BBW80_01485	BBP28_06055	BBP27_04515	Puncturing device inner tube
TssE	BBW80_02630	†	BBP27_04525	T6SS baseplate component
TssF/ImpG	BBW80_02645	BBP28_07375	BBP27_04530	T6SS baseplate component
TssG	BBW80_02640	BBP28_06155	BBP27_04535	T6SS baseplate component
TssH/ClpB	BBW80_01480	BBP28_06150	BBP27_04540	AAA ATPase
TssI/VgrG	*	BBP28_06090	BBP27_04565	Puncturing device tip protein
PAAR	BBW80_01515	BBP28_06085	BBP27_04575	PAAR domain-containing protein
TssJ	BBW80_02635	np	BBP27_04545	Outer-membrane lipoprotein
TssK	BBW80_01500	BBP28_08095	BBP27_04550	T6SS baseplate component
TssL	BBW80_01495	np	BBP27_04555	Inner membrane, 1 TM, membrane complex
TssM/IcmF	BBW80_02655	BBP28_06095	BBP27_04560	Inner membrane, 3 TMs, membrane complex

*Up to four VgrG with specific effector immunity (EI) pairs are predicted to be within isolate KU1003-01.

†CDS is present across the ends of two contigs.

np, not present.

To expand this discovery to the other isolates, Mauve alignment and nucleotide blastn homology searches were conducted for each of the T6SS components. These revealed that KU1003-02 and RH3002v2g also possess homologues of the T6SS. However, the T6SS in these isolates is different from that seen in KU1003-01. Confirmation of these two different T6SS types was achieved through amino acid sequence alignments using clustal Omega (data not shown). Annotations for T6SS functions were confirmed against the SecRet6 database [46]. No T6SS was identified in isolate RH3002v2f (* N. cinerea *), and therefore the T6SS was only found here in the isolates identified as *N. subflava* biovar *perflava*. Analysis of 15 draft and complete *N. subflava* genome sequences in the NCBI database indicated that the majority of these (8 out of 15) possess T6SSs. The most commonly identified T6SS was the type identified within isolates KU1003-02 and RH3002v2g, with 5 out of 15 genome sequences from the database that were analysed possessing this type and the remaining 3 matching KU1003-01.

The T6SS in isolate KU1003-01 appears to have all of the 13 core components necessary to produce a functioning system [121]. These were identified on putative genomic islands at two different loci with *tssE*, *tssJ*, *tssG*, *tssF*, *tssA,* and *tssM* being identified in one cluster and *tssH*, *tssD*, *tssL*, *tssK*, *tssB*, *tssC*, *vgrG,* and PAAR at a separate locus. Isolate KU1003-01 is predicted to have up to four *vgrG* each with different effector–immunity (EI) pairs. The T6SS identified in isolates KU1003-02 and RH3002 v2g, by comparison, were located at a single locus and were predicted to have only one *vgrG*.

Bioinformatic studies have so far identified the T6SS in around 25 % of Gram-negative bacteria [121] and the products of the genes involved in its assembly are evolutionarily well conserved [122]. It is believed that one function of the T6SS is to aid bacteria in successful colonization and survival within competitive niches and it has been proposed that this system is responsible for shaping the composition of microbial populations [123–125].

It is likely that naturally competent *Neisseria* spp. colonizing the same niche acquire DNA from one another [12] and the T6SS has been shown to aid this process. T6SS-positive species have been shown to acquire new effector–immunity pairs from their neighbours through this mechanism [126]. In species other than *Neisseria *, the T6SS has also been shown to play a role in nutrient acquisition [127].

It is possible that the co-isolated KU1003-01 and KU1003-02 have shared T6SS immunity genes, which has allowed them to co-exist together, although further investigation is needed to determine if this is the case. By carrying out further study into the mechanisms of the T6SS, novel therapeutic interventions could be developed with regard to pathogen-related infections. One possible intervention, as suggested by Unterweger *et al*. [128], would allow non-pathogenic commensal bacteria such as these *N. subflava* biovar *perflava* possessing the T6SS to outcompete pathogens such as *N. meningitidis* and *N. gonorrhoeae* within a specific niche. The T6SS effector proteins, which are antibacterial to competitor species, have also been proposed to be developed as therapeutic agents against multidrug-resistant bacterial pathogens [129].

### Similar iron acquisition systems to those of the pathogens, as well as systems not previously seen in *Neisseria* spp.

In order to establish an infection, pathogenic bacteria must be able to obtain iron from a host and in the case of the pathogenic *Neisseria* spp. it is considered to be a major virulence determinant [8–11]. It is believed that diversity in iron uptake genes aids colonization of different *Neisseria* spp. within the same niche where host antibodies are targeted towards a variety of iron acquisition components from different bacterial species [12].

Isolate RH3002v2f was found to contain CDSs homologous to *fbpA*, *fbpB,* and *fbpC* for an ABC-type Fe3+ transport system (FbpABC) and *lbpA* and *lbpB* for lactoferrin-binding proteins (Table 3), which were not present in the other isolates. CDSs with homology to *hmbR* were identified in isolates KU1003-01, KU1003-02, and RH3002v2g for haemoglobin receptor (Table 3). This specificity in differential iron utilization for the *N. subflava* biovar *perflava* isolates versus the *N. cinerea* isolate agrees with earlier results [12].

### A two-partner secretion system, not previously identified in *Neisseria* spp

While none of the isolates were determined to be haemolytic, a two-partner secretion system (TPSS) was identified within isolate RH3002v2f that was predicted to encode a putative haemolysin secretion system (Table 4). This system could not be identified in any of the other isolates. This TPSS is similar to the ShlA/ShlB system of *Serratia marcescens* [130], which only secretes its haemolysin in low-iron conditions [131], which may explain our observations here. In *S. marcescens *, *shlA* encodes a haemolysin and *shlB* an outer-membrane protein required for the secretion and activation of ShlA [130]. The TPSS identified in isolate RH3002v2f has orthologues in a very small number of other commensal neisserial genomes, including *N. cinerea* [53]. Many pathogens, including *N. meningitidis* and *N. gonorrhoeae *, are known to possess a wide range of mechanisms for iron acquisition [11] and are able to utilize haem released by haemolysis as an iron source. Heme constitutes the largest source of iron within a human host [132] and while there is normally a limited supply within the nasopharynx, meningococci are thought to be able to take up small amounts through the expression of TonB-dependent receptors [133].

### A heme system, not previously identified in *Neisseria* spp.

An operon containing a TonB-dependent haem acquisition and utilization system was identified within the genome for isolate RH3002v2g, similar to the *hutWXZ* system in *Vibrio cholerae* [134]. The same system was also identified in the genome sequence data for isolate KU1003-01, as well as in the 15 *N. subflava* spp. investigated and in a number of *N. perflava *, *N. flavescens *, *N. elongata,* and *N. lactamica* genomes. Within *N. subflava *, the genes surrounding the TonB-dependent haem acquisition and utilization system fell into two groups, consistent with the type of T6SS they possessed.

In the first group, the TonB-dependent haem acquisition and utilization system was identified next to CDSs encoding a T6SS VgrG protein with a predicted anti-eukaryotic effector. T6SS effects have previously been shown to be involved in metal acquisition [135] in *Yersinia pseudotuberculosis* and iron acquisition in *P. aeruginosa* [125]. It is possible that under conditions of iron starvation, the T6SS and TonB-dependent haem acquisition and utilization systems act together for the purpose of iron acquisition in some of the commensal *Neisseria* spp. In the second group, which consisted of the second neisserial T6SS type, as well as all T6SS-negative *N. subflava *, the haem acquisition and utilization system is associated with a zonula occludens toxin-like protein (Zot) homologue; Zot disrupts mucosal tight junctions in *V. cholerae* and orthologues have been found in *N. meningitidis* and *Campylobacter concisus* [136, 137].

## Conclusions

In-depth investigations of the genome sequences of non-pathogenic *Neisseria* spp. are of interest in their own right, revealing themselves to not only be reservoirs of a large gene pool for the naturally competent genus, but also to contain genetic features not previously seen, such as the first reported *Neisseria* T6SS. A wealth of new biological insight into this genus can be gained by further investigating the functions of the previously unexplored features described here in this *N. cinerea* and its TPSS and three *N. subflava* biovar *perflava* and their T6SSs. In addition to the potential of the antibacterial activity of the T6SS expressed by these isolates, the individual T6SS effectors identified in these genome sequences might also be promising avenues for development of antibacterials against multidrug-resistant pathogens.
